# Robotic Surgical Procedures for Ventral Hernia Repair

**DOI:** 10.3389/jaws.2025.14212

**Published:** 2025-02-13

**Authors:** M. W. Christoffersen, K. Andresen, Helene Perregaard, N. A. Henriksen

**Affiliations:** ^1^ Department of Surgery, Zealand University Hospital Køge, Køge, Denmark; ^2^ Department of Surgery, Copenhagen University Hospital - Herlev and Gentofte, Herlev, Denmark; ^3^ Department of Surgery, Center for Perioperative Optimisation, Copenhagen University Hospital - Herlev and Gentofte, Herlev, Denmark; ^4^ Department of Surgery, Nordsjællands Hospital University of Copenhagen, Hillerød, Denmark

**Keywords:** robot-assisted ventral hernia repair, ventral hernia, TARM, E-TEP, RoboTAR

## Abstract

The recent availability of robotic platforms has facilitated the adoption of advanced minimally invasive ventral hernia repair. Robotic-assisted ventral hernia repair is an evolving field with many new techniques and acronyms for different accesses and approaches. This paper aims to describe the four currently most used procedures for robotic ventral hernia repair, all of which are MIS sublay repairs; robotic Trans-Abdominal-Preperitoneal (r-TAPP), robotic Trans-Abdominal-Retromuscular-Mesh (r-TARM), robotic Extended-Totally-Extra-Peritoneal (r-E-TEP), and robotic Transversus-Abdominis-Release (RoboTAR). Their descriptions are supported by illustrations. The paper describes trocar placement, practical tips and tricks, and briefly discusses the indications for each procedure. Furthermore, technical details such as the incision of the flap, access to the correct anatomical planes, dissection techniques, handling of the hernia sac, mesh choice and placement, and restoration of the abdominal wall layers are described. In conclusion, robotic ventral hernia repair has gained wide acceptance with promising postoperative results. There are many different techniques and approaches available, and this paper describes the four most commonly performed procedures in a detailed step-by-step fashion.

## Introduction

Primary ventral and incisional hernia repairs represent high-volume procedures performed electively as open or minimally invasive surgery (MIS). Open repairs carry a substantial risk of wound-related complications, especially in obese patients which increases short-term morbidity, and hospital costs, along with the risk of recurrence [1–3]. Three decades ago, laparoscopic intraperitoneal onlay mesh (IPOM) repair was introduced as an MIS alternative to open repair and was shown to be superior in terms of reducing wound complications and, suggestively, even recurrence [4, 5].

In laparoscopic IPOM repair, an intraperitoneally coated mesh is fixated to the abdominal wall with tackers and/or transfascial sutures [6]. Unfortunately, rare but severe complications, such as intestinal adhesions to the mesh occasionally can lead to small bowel obstruction and adhesions cancomplicate future surgery [7, 8]. Additionally, acute and chronic pain due to transfascial mesh fixation devices has made the IPOM approach less popular in recent years [9]. The optimal plane for mesh placement has been discussed widely, however, placing the mesh in the preperitoneal and retro rectus planes appears to be associated with fewer complications and is recommended in international guidelines [10, 11]. Several laparoscopic procedures such as ventral Trans-Abdominal-PrePeritoneal (TAPP), extended Totally-Extraperitoneal (E-TEP), endoscopic Mini- or less-open sublay (eMILOS) [12–14] have been developed to replace the IPOM technique with the purpose of keeping the mesh out of the intraperitoneal cavity. Unfortunately, the adoption of these laparoscopic techniques has been limited due to a steep learning curve and high technical requirements, leading many surgeons to revert to traditional open procedures [9, 15].

The increased availability of robotic platforms has facilitated the adoption of minimally invasive techniques with mesh placement in the preperitoneal and retro rectus planes. The robotic platform provides increased degrees of freedom and precision of movement for the surgeon. This is possible via a stable and ergonomic platform, elimination of physiologic tremor, and three-dimensional visualisation [16]. The articulation of the robotic arms has improved both dissection and suturing. Furthermore, this technology has made component separation techniques, such as transversus abdominis release (TAR), accessible through minimally invasive approaches, enabling even large incisional hernia repairs to be performed with an MIS approach. Early results are promising, showing fewer wound infections, less postoperative pain, and shorter hospital stays compared to open techniques [17–19]. However, long-term results are pending.

The aim of this paper is to describe the current indications and surgical details of the four most frequently used procedures for robotic ventral hernia repairs all of which are MIS sublay repairs; robotic Trans-Abdominal Preperitoneal (r-TAPP), robotic Trans-Abdominal-Retromuscular-Mesh (r-TARM), robotic Extended-Totally-Extra-Peritoneal (r-E-TEP), and robotic Transversus-Abdominis-Release (RoboTAR). Table 1 is describing limitaions and advantages of the four different procedures.

**TABLE 1:** Summary of advantages and limitations for each procedure.

Procedure	Advantages	Limitations
r-TAPP	Good view of the abdomen, easy access, saves the retromuscular plane, easy to perform in fatty rich areas, no anatomical boundaries, does not alter the contour of the abdomen	Dissection can be difficult in very thin peritoneum found in the area of the lateral part of the rectus, difficult to use if the peritoneum is thin or injured, or in medium to large-sized incisional hernias
RTARM	Good view of the abdomen, easy access, easy to start with, can be used for most hernias	Difficult suturing the flap close to the camera, incising an otherwise healthy posterior sheath. May alter the contour of the abdomen, risk of damage to neurovascular bundles
rE-TEP	No flap opening, no flap closure, can be used for most midline hernias	Steeper learning curve, more difficult access, risk of iatrogenic intestinal lesion with adhesions to the hernia sac, may alter the contour of the abdomen, risk of damage to neurovascular bundles
RoboTAR	Can be used for even large incisional hernias, easy transabdominal access	Long operative time, re-docking, risk of damage to neurovascular bundles

### General Considerations for Robotic Ventral Hernia Repair

#### Patient Placement and Operating Room Set-Up

**FIGURE 1:**
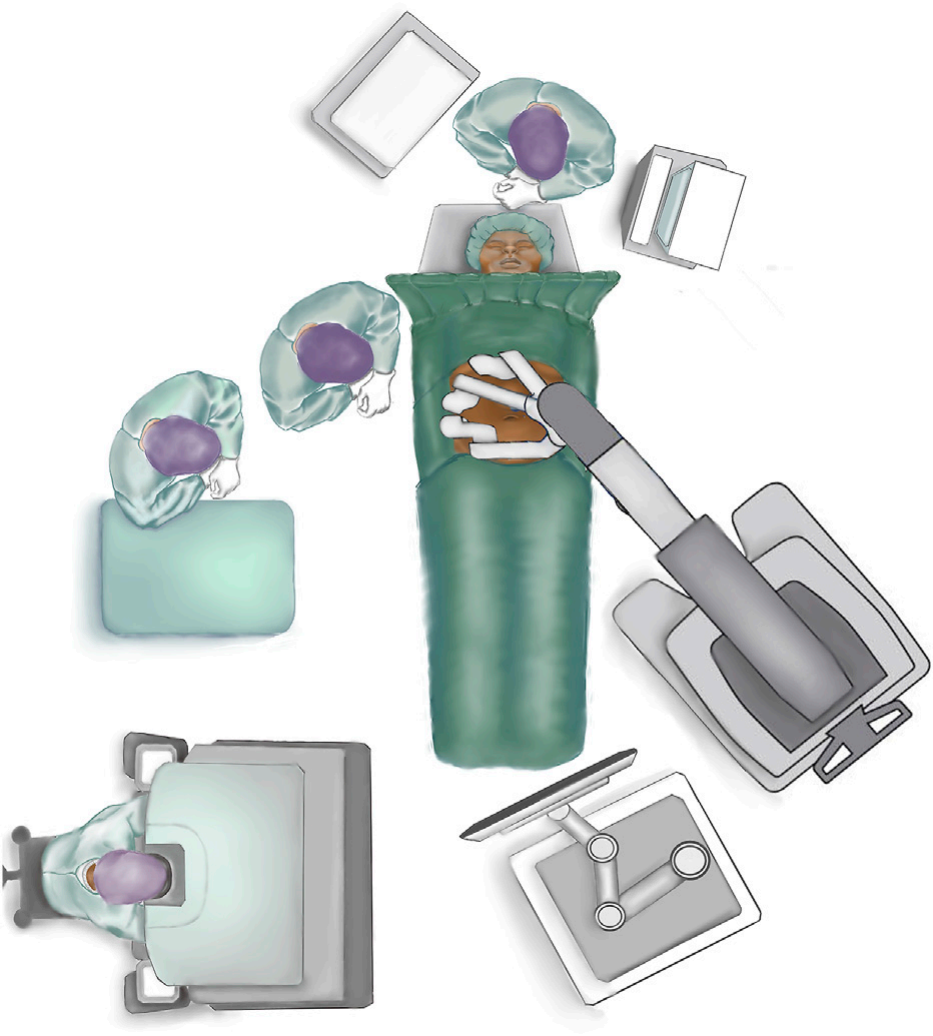
Patient placement and operating room set-up.

The patient is placed in a supine position close to the border of the table on the side where trocar placement is planned (if lateral docking) (Figure 1). The arm at the side of the trocar placement is tucked into the patient’s side. Both arms are tucked in if docking from both sides is planned (double docking). The table is flexed at the point of the hip to increase the distance between the costal margin and the anterior superior iliac spine. If the table does not have the hip flexion mode, the same dorsal flexion can be obtained with 20–30° “legs down” and a slight Trendelenburg. This can be done with the patient awake to ensure patient comfort in this hyperextended position. It is advisable to use a face and tube protector [20, 21].

#### Anaesthesia

The patient is anaesthetised and must be completely relaxed using neuromuscular blockage during the entire time the robotic platform is docked to the patient. A urinary catheter is placed if the procedure is longer than 2 hours. According to guidelines, perioperative antibiotics (1,500 mg Cefuroxime) are administered [11, 20, 21].

#### Trocar Placement

**FIGURE 2:**
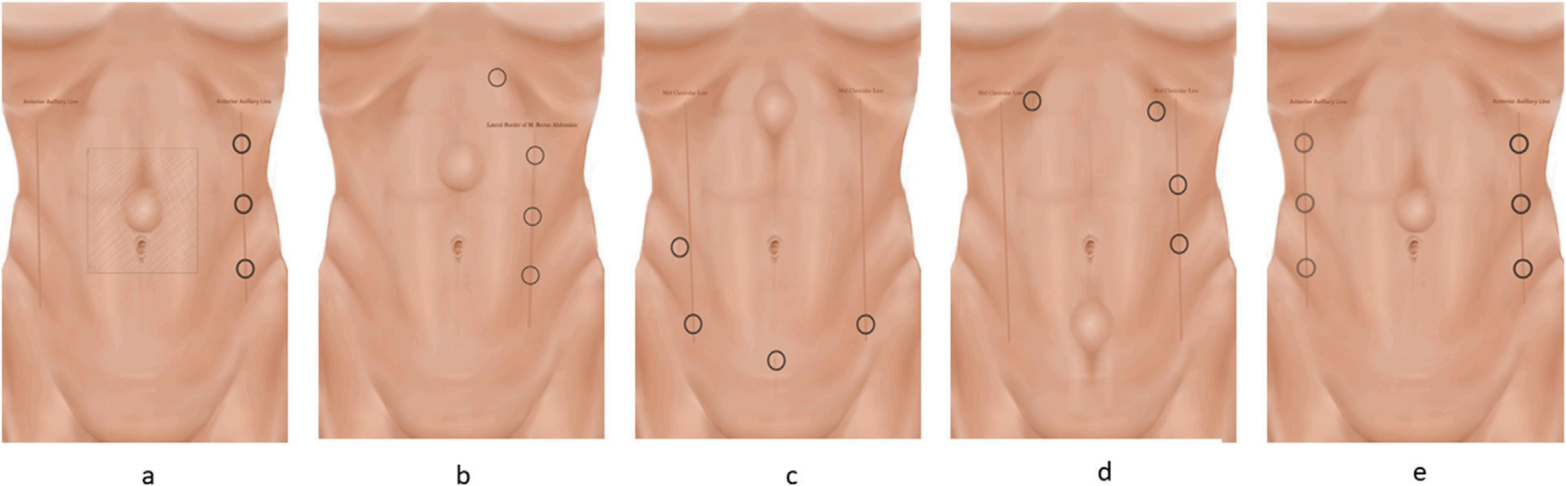
The different trocar placements. The trocars are placed on a line. The camera trocar is always placed in the middle, with the scissors in the dominant hand and the grasper in the non-dominant hand. Ensure a minimum of 8 cm between the three trocars. Trocar placement for the E-TEP procedure is described in the text. **(A)** Lateral docking for TAPP and TARM. **(B)** Lateral docking for E-TEP with subcostal access and helping trocar. **(C)** Suprapubic docking for E-TEP. After cross-over, the robot is re-docked. **(D)** Top docking for E-TEP. After cross-over, the robot is re-docked. **(E)** Bilateral TAR docking.

The majority of procedures can be performed with three robotic trocars. A rule of thumb is that the robotic trocars are optimally placed with a 15–20 cm distance, or as far as possible, from the hernia defect for the transabdominal approaches, and with a minimum of seven to 8 cm between each trocar [20, 21]. Trocar placement is guided by the location of the defect and previous abdominal surgeries. The majority of midline hernias can be approached with a lateral docking. If the patient has a narrow waistline circumference or in cases of very cranially or caudally located defects, suprapubic or cranial docking is preferred (Figures 2A–F). In the initial phase, ultrasonographical guided drawings of the borders of the recti muscles, the linea alba, and the defect may help avoid injury to critical neurovascular structures. Furthermore, it may be helpful to draw the margins of the proper mesh size and mark the incision points of the posterior rectus sheath with transcutaneous needles.

#### Instruments

All robotic ventral hernia procedures can be performed with a 30-degree camera, one bipolar grasper, one monopolar scissor, and a needle driver.

#### Access to the Intraabdominal Cavity, Flap Development, and Dissection Techniques

Are described specifically under the different procedures.

#### Hernia Sac Dissection

A key principle of hernia sac dissection is to “encircle the enemy” – which means that the sac is easier to dissect and reduce if the areas cranial and caudal to the defect are dissected first. The hernia sac should be reduced completely if possible. If the sac is very thin and fragile or large and spiculated, it may not be possible to reduce it. In these cases, the hernia sac must be cut and detached from the edges of the fascial defect and the hole in the posterior layer must be closed. In this case, it is necessary to recruit more coverage by dissecting the peritoneum further from the defect to be able to close the hole in the peritoneum.

#### Closure of the Defect and Plication of the Linea Alba

The hernia defect should always be closed with a running suture. Both absorbable and non-absorbable sutures are used and there is no evidence to support one or the other. The pneumoperitoneum must be reduced to 10–8 mmHg or less when the defect is closed. The defect can be closed transversely if there is only one defetct and it is small (>3 cm) it can be closed in a ttransverse direction. In cases of multiple defects along the linea alba or significant diastasis recti plication of the entire linea alba with a running barbed suture like V-LOC^®^ or Stratafix^®^ is performed by placing the sutures on either side of the linea alba itself. A small part of the anterior fascia should be left when performing the cross over (see later in the manuscript). After reducing the intraabdominal pressure to 10–8 mmHg or less, the sutures can be tightened with the “shoelace” technique. It is advisable to run the suture back and forth to ensure the strength of the closure. In cases of a wide diastasis the plication may be done with the inverted plication suture technique to prevent bulging of the suture line. In the case of a large hernia sac it is advisable to reduce the dead space by putting sutures into the hernia sac (if not reduced). The umbilical “innie” can be re-established by placing an absorbable Vicryl suture stitch in the subdermal skin of the umbilicus and attaching it to the underlying fascia.

#### Mesh Choice and Fixation

A permanent, macroporous, synthetic, non-coated mesh is recommended for preperitoneal or retromuscular placement [10, 11]. The mesh should be fitted to the size of the entire dissected area. Measurement of the retro rectus/preperitoneal space is performed with a surgical ruler to be able to fit the mesh to the correct size. The mesh is then fixated with sutures up against the anterior abdominal wall or using a self gripping mesh. In E-TEP and RoboTAR procedures the mesh is placed flat against the posterior sheath without fixation or using fibrin glue.

### TransAbdominal PrePeritoneal (TAPP) Repair

In the TAPP procedure the dissection and mesh placement is in the preperitoneal plane, which is the innermost layer of the abdominal wall. It is important to know the structures and anatomical features of the peritoneum in the different parts of the abdominal wall to perform a safe and sufficient dissection. The ventral TAPP technique can be used for various hernias and defect sizes and placements, but it can be difficult if the peritoneum is thin, injured, or violated. The key elements of this technique are taken from the TAPP technique used for inguinal hernias [22]. The aim of the TAPP repair is to place the mesh anteriorly to the peritoneum, thus avoiding contact between the mesh and intraabdominal organs, but this requires a robust preperitoneal layer. Although there is no real defect size limit for this surgical technique, it may be easier in primary hernias, smaller defect sizes, “out of midline” defects or defects located in the cranial or caudal part of the midline. One of the key principles of ventral TAPP repair is that “fat is your friend”: meaning that the dissection of the preperitoneal plane is easier in areas that are rich in preperitoneal fat. Thus, this technique can be more challenging in very thin patients.

This technique is useful for small- to medium-sized primary ventral hernias, subxiphoid hernias, or primary lumbar hernias [23]. The preperitoneal plane can be difficult to use in incisional hernias or recurrent hernias.

It is advisable to use the TAPP technique whenever possible to preserve the retromuscular plane, saving the retromuscular plane for possible recurrent repairs, or if the patient is operated on later and has another hernia.

Common complications of the TAPP and TARM techniques are over looked peritoneal lesions or tearing of the closure of the posterior layer, resulting in small bowel obstruction. To avoid this, all holes and flaps must be thoroughly closed, and it should be ensured that the dissected flap is large enough to prevent “tear outs” caused by excessive tension in the closure of the flap.

**FIGURE 3:**
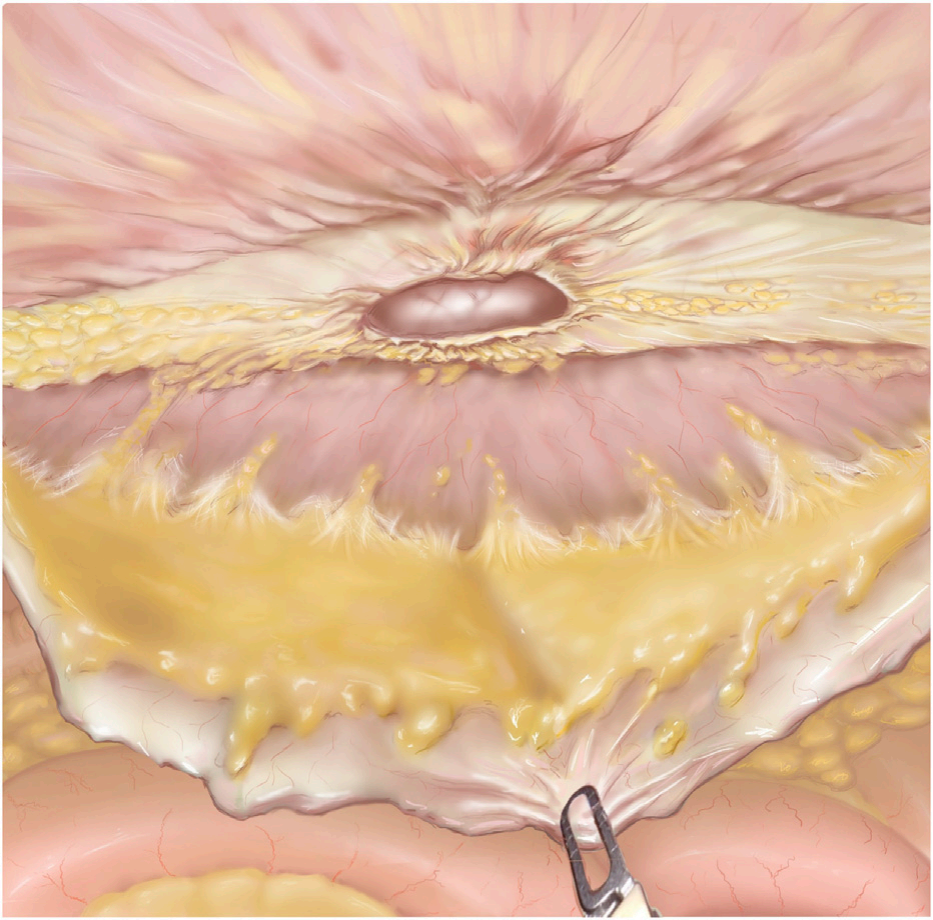
TAPP after flap development. The hernia defect is seen in the middle of the figure. The grasper is pulling down on the preperitoneal flap. The linea alba is mostly cleared of fibro-fatty tissue.

**FIGURE 4:**
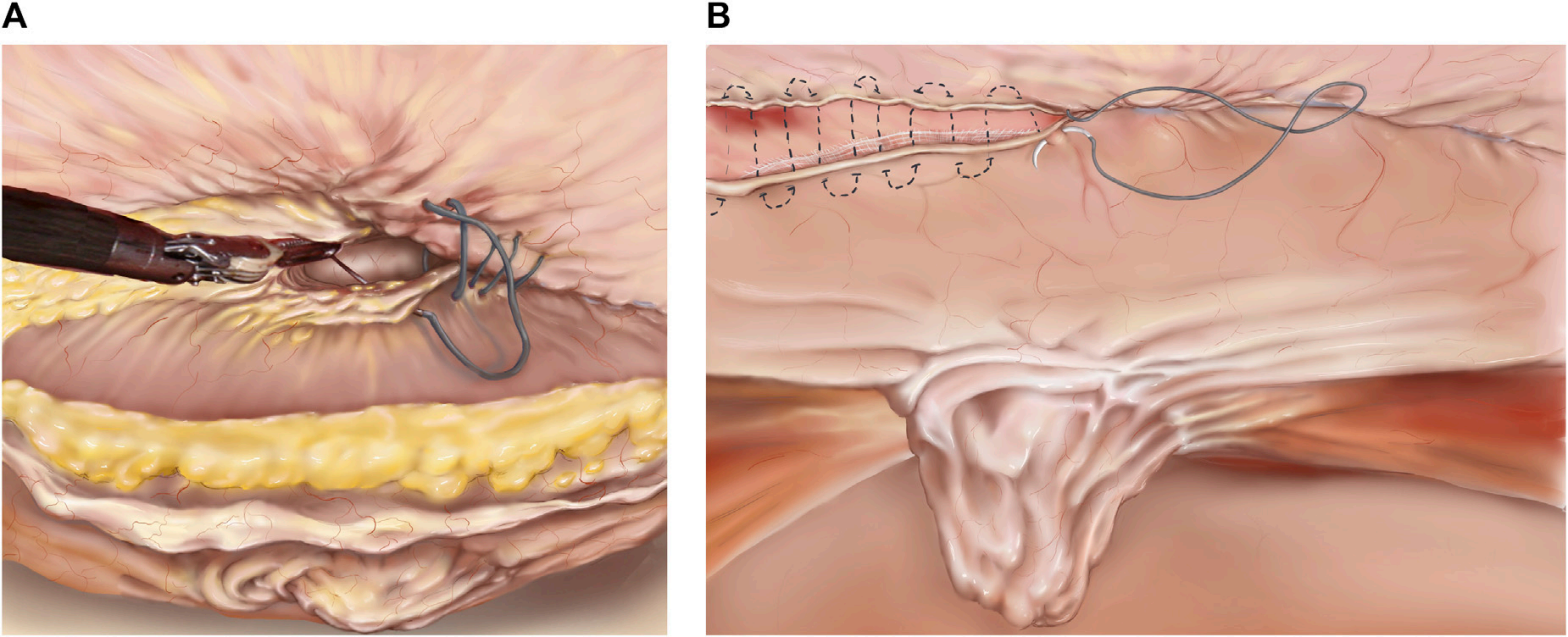
**(A)** TAPP/TARM. Suturing the defect. The defect is closed with a barbed suture in a cranial to caudal direction. The suture is started cranially to the defect and continued caudally to the defect. In the lower part of the figure, the completely reduced hernia sac is seen. **(B)** TAPP/TARM. Closing the flap. The flap, consisting of peritoneum in the TAPP or peritoneum and retro rectus sheath in the TARM is closed with a running barbed suture. In the case of a thin or fragile flap, a “dolphin” suture may be used as illustrated by the dotted line. The completely reduced hernia sac can be seen in the middle of the figure.

1. Access to the abdominal cavity and trocar Placement: Pneumoperitoneum to 12 mm Hg can be obtained with a Veress needle at Palmer’s point. Three robotic trocars are used. For defects in the midline/mid-abdomen trocar placement is optimally placed lateral to the border of the rectus sheath (Figure 2A) [24]. However, trocar placement may differ if the hernia is in the flank, epigastrium, or pubic area as described above.
2. Flap Development: Depending on the size of the hernia defect, a preperitoneal flap is developed from a peritoneal incision at a proper distance from the hernia defect. The flap must be large enough to cover the entire mesh. It may be helpful, before starting the dissection, to draw the size of the mesh on the skin of the patient and have the bedside assistant mark the corners with a needle [25, 26]. When starting the creation of the flap it is advisable to start in an area rich in preperitoneal fat. The flap must be grasped gently with the bipolar grasper close to the area of dissection and pulled towards the operating surgeon - not downwards. At this point, gentle push must be applied upwards with the closed scissors onto the posterior sheath, and the small vessels should be cauterised. Pressure should never be applied directly to the peritoneum. It can sometimes be helpful to “dig a cave” away from the camera/instruments and then extend it gently in all directions. This will sweep the peritoneum away from the overlying fascia. Care should be taken not to injure the posterior rectus sheath, the linea alba above, or the peritoneum. In the lateral and lateral-cranial part of the abdomen it is also advisable to recruit the transversalis fascia to obtain a stronger posterior layer. Tears or rents in the peritoneum created during dissection must be closed afterwards. It should be noted that the peritoneum has two layers, which is most evident in the caudal part of the abdomen. In the areas of a very thin peritoneum it is essential to recruit all layers including the fascia transversalis [27].
3. Hernia Sac Reduction, facial defect closure, and mesh placement: As described above.
4. Preperitoneal Flap Closure: After the flap has been developed (Figure 3) the defect is closed with a size 0 or 1 running barbed suture (Figure 4A). It is a matter of debate whether a long-term absorbable or non-absorbable suture should be used to close the defect. A synthetic mesh is then placed, and the peritoneal flap is closed to exclude the mesh from the intraabdominal cavity with a running absorbable barbed suture. A regular “over-and-over” suturing can be used but in cases of fragile peritoneum a “dolphin” suture technique is advisable (Figure 4B).

### TransAbdominal RetroMuscular Repair (TARM)

Robotic-assisted placement of a mesh in the retromuscular space has different names, one of which is “Transabdominal Retromuscular Umbilical Prosthetic” (TARUP) hernia repair [26], however, the term transabdominal retromuscular repair (TARM) may be more accurate [28, 29]. The TARM repair uses the retromuscular plane laterally and the preperitoneal plane in the midline. This means that the technique involves a “cross-over” where the two retro rectus spaces are combined with the mid-preperitoneal plane posterior to the linea alba, to form one large, connected space. This approach is primarily used for midline ventral hernias, but also for more laterally placed defects, i.e., previous stoma-site hernias. Indications for this procedure include medium to large -sized (<3 cm) primary ventral hernias [23], all incisional hernias, hernias with concurrent large diastasis, multiple defects or Swiss cheese, or as a “bail out” of a TAPP repair if the peritoneum proves to be too thin or disrupted.

While not a true complication, it is worth noting that both the TARM and E-TEP procedures may alter the contour of the abdominal wall by cutting the medial aspect of the posterior sheath. Also, plication of the linea alba may result in a bulge or retraction of the skin. This can be especially evident in slender patients. These concerns should be discussed with the patients preoperatively.

**FIGURE 5:**
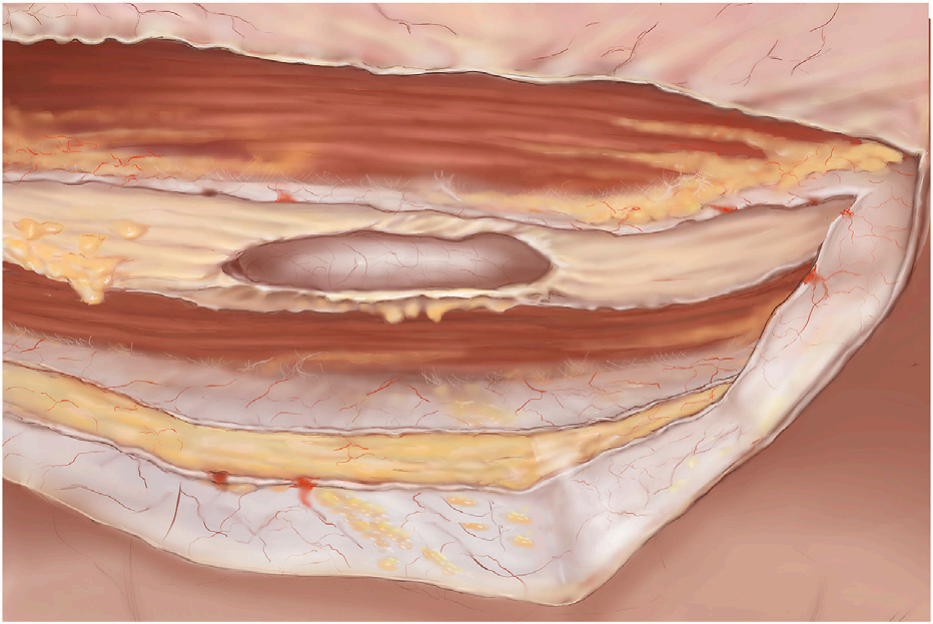
TARM. Dissected retromuscular and preperitoneal space. The cut lateral border of the ipsilateral retro rectus sheath is visible. In the midline both the right and left medial borders of the retro rectus sheath can be seen cut and contralaterally, the retro rectus space is visible. Both rectus muscles are visible and the linea alba is cleared of fibro-fatty tissue.

1. Access to the abdominal cavity and Trocar Placement: Access to the abdominal cavity and establishment of pneumoperitoneum is performed as described above. Three robotic trocars are used. The trocars must be placed on a line lateral to the lateral border of the rectus sheath. The trocars may be placed elsewhere depending on the location of the defect. This paper describes the lateral approach that can be used for the majority of midline defects (Figure 2A).
2. Flap Development: Development of the retromuscular flap entails incising the lateral border of the posterior rectus sheath starting on the ipsilateral side to the instruments and camera, approximately 5-6 cm lateral to the linea alba [25, 26]. Care must be taken not to injure the inferior epigastric vessels or the neurovascular bundles, both of which are located in the lateral part of the posterior rectus sheath. Once the posterior sheath is incised parallel to the linea alba, the flap of the posterior sheath is grasped, and the rectus muscles are gently swept upwards from the posterior sheath. It is important to clear the posterior rectus sheath completely from fibrofatty tissue and cauterise small vessels to prevent the development of a postoperative haematoma. After completing the lateral to medial retro rectus dissection on the ipsilateral side, the medial border of the posterior rectus sheath is identified by visualisation of the fibres of the posterior rectus sheath going in a vertical direction.
3. Midline Cross-over: An incision 1-2 cm lateral to the medial border of the posterior rectus sheath is performed. The incision must start away from the defect in the cranial or caudal part. The incision should immediately reveal the yellow preperitoneal fatty tissue of the preperitoneal plane posterior to the linea alba. Care must be taken not to cut upwards, thus injuring the linea alba, or too low, resulting in injury to the posterior layer. Once this access is made, the preperitoneal space is developed along the midline. The dissection technique is a combination of sweeping down the fat and clearing the linea alba of fatty tissue. The fat stays on the peritoneum. In incisional hernias there may be scarring along the area of the previous incision. In the umbilical area several vascular circuits can be found, and care should be taken to cauterise all perforator vessels from the inferior epigastric and subdermal plexuses to obtain haemostasis [25]. Also, the umbilical ligaments need to be cut to clear the entire space. Notably, the linea alba is typically very narrow and may almost be non-visible caudal to the umbilicus.
4. Hernia Sac Reduction: As described above.
5. Contralateral rectus sheath dissection: Once the midline crossover and preperitoneal dissection are completed in both the cranial and caudal directions to the extent that allows for a 5 cm mesh overlap, the medial border of the contralateral posterior rectus sheath is visualised through the peritoneum as a darker shade compared with the linea alba. The crossover is now continued to the contralateral side through an incision 1-2 cm contra lateral to the linea alba. Red muscle fibres should be visible, and the incision of the contralateral rectus sheath is continued in a cranial and caudal direction. It is important to leave a small part of the anterior fascia when cutting the posterior sheath when performing the crossover (Figure 5). The contralateral retro rectus space is dissected as described above, dissecting laterally until the lateral edge of the rectus sheath is reached. Care must be taken not to injure the neurovascular bundles.
6. Cranial dissection: It is important to be familiar with the anatomical aspects of the subxiphoid area (defined as starting 6 cm below the xiphoid process) to preserve the neurovascular bundles that enter the retro rectus space much more medially in the cranial part of the abdomen, but also to prevent damage to the pars abdominalis of the diaphragm. It is advised to switch from the retro rectus space to the preperitoneal space in the cranial part of the abdomen to avoid neurovascular injury. This is done by cutting the posterior sheath in a transverse fashion starting 6 cm below the xiphoid process and entering the preperitoneal space. If going further cranially, care must be taken not to injure the pars abdominalis of the diaphragm. The dissection in the midline must be completely preperitoneal leaving the fatty tissue (fatty ball or trident) on the xiphoid process [30].
7. Fascial defect closure, mesh placement, and flap closure: As described above.

### Extended Totally Extraperitoneal (E-TEP) Approach

The E-TEP repair is also a retromuscular repair technique. The extended Totally Extraperitoneal (E-TEP) approach builds upon the principles of TEP for inguinal hernias but extends its application to a wider range of hernias, such as medium- and large ventral and incisional hernias [12]. Some of the advantages of this technique lie in the direct access to the retromuscular space without the necessity of incising the lateral edge of the ipsilateral posterior sheath. Another advantage of the E-TEP technique is the elimination of the flap closure, which can present a challenge, because of the proximity to the trocars. Data is sparse regarding the best approach but outcomes seem to be equivalent [29, 31, 32].

Common complications of the E-TEP procedure, especially when on a learning curve, are damage to the neurovascular bundles during trocar placement at the lateral border of the posterior sheath. Another complication can be an iatrogenic intestinal lesion when dissecting the hernia sac. This can be avoided by performing laparoscopy including adhesiolysis if necessary, before the E-TEP procedure, as described below.

1. Laparoscopy/“peek inside”: In all incisional or ventral hernia repairs where intestinal adhesions are expected on preoperative CT scans it is advised to start transabdominally and lyse intestinal adhesions under direct vision to avoid iatrogenic intestinal lesions while dissecting the hernia sac or posterior elements.
2. Access to the Retro rectus Space: Access is guided by the location of the hernia and previous abdominal surgeries. Generally, it is optimal to start developing the retro rectus space away from the hernia sac and scarred areas. The majority of midline hernias can be approached from a lateral dock. In cases of cranial or caudal located defects suprapubic- or cranial docking is preferred.
  Lateral Docking (Figure 2B): The lateral access can be used for the majority of defects but can be difficult if the patient is slender and/or has a narrow waistline (See below). Access to the retro rectus space is most often easily achieved by a “pre-costal access” to the left or right retro rectus space as previously described [12]. An incision is made approximately 2 cm caudal to the lower costal margin and approximately 5-6 cm lateral to the midline depending on the width of the midline, due to diastasis or previous surgery. A horizontal incision of the skin is made, and the anterior sheath can be incised under direct vision. The retro rectus space can now be entered either bluntly with the camera in the 5 mm trocar- or with a 12 mm trocar or under direct vision. This is easiest done with a 0-degree, 5 mm scope but can also be performed with the 30°, 8 mm robotic scope. The aim is to have the white, posterior sheath at the bottom and the red muscle fibres at the top. The space is further developed using carbon dioxide insufflation (12 mmHg) and the camera, pushing gently in a caudal direction. Additional, careful, sideways sweeping movements under direct vision may be made to further dissect the space. Avoid injury to small vessels, since bleeding will quickly obscure vision in the narrow space. Oftentimes a laparoscopic hook or Harmonic scalpel can be used to cauterise small vessels to make space for placing additional trocars on a line downwards. Additional robotic trocars can be inserted under direct vision in the most lateral part of the retro rectus space to avoid injury to the neurovascular bundles.
  Suprapubic docking (Figure 2C): This access is used for cranially located defects, if the patient is slender and/or has a narrow waistline. A horizontal incision 2 cm below the umbilical line and just medial to the lateral border of the rectus sheath is made. The anterior sheath must be open and the retro rectus space developed cranially and caudally using carbon dioxide insufflation and pushing movements with the scope under direct vision as described above. Upper midline defects require at least two additional ports: one 3 cm above the pubic symphysis in the left paramedian line and another 2 cm above it in the right paramedian line. This approach can be challenging if the patient has had previous surgery in the lower part of the abdomen i.e., a Pfannenstiel or lower midline incision.
  Top docking (Figure 2D): The same initial access as described for lateral docking must be used. One or two additional trocars should be placed further caudally on the abdomen and dissection and the crossover should be performed in the cranial part of the abdomen. An additional trocar is then placed in the upper right retro rectus space, the robot is re-docked, and the dissection is carried out in a cranial to caudal direction (see below).
3. Retro rectus Dissection:
  Lateral approach: The ipsilateral retro rectus space is dissected; crossover and contralateral retro rectus dissection are performed as described above for TARM crossover.
  Suprapubic docking: The two retro rectus spaces are combined by incising the posterior rectus sheaths on both sides (cross-over) in a cephalad direction, connecting the retro rectus spaces and the preperitoneal space in the midline. See above for “cranial dissection” [30].
  Cranial docking: The ipsilateral retro rectus space should be dissected from cranial to caudal. Then, the crossover should be performed as described above, after returning to the upper abdomen. A fourth trocar must be placed at the level of the first trocar on the right paramedian line for bilateral dissection. The three spaces should be dissected until the pubic bone is visible in the midline and the lateral aspect of the posterior rectus sheath is visible [22].
4. Hernia Sac Reduction: As described above.
5. Fascial Defect Closure: As described above.
6. Mesh placement: As described above.
7. Pneumoperitoneum Release: To release the pneumoperitoneum gradually, ensuring that the mesh remains flat and properly extended.

### Robotic Transversus Abdominis Release (RoboTAR)

The open posterior component separation or transversus abdominis release (TAR) technique not only enables the advancement of the posterior sheath for tension-free closure, but also allows a large mesh overlap, and thus has revolutionised hernia repair [33]. The TAR technique is applicable to robotic hernia repair and can be performed either transabdominally or as an E-TEP approach [20, 34]. The classical approach is transabdominal with lateral access, double docking and an E-TEP technique. Both suprapubic and cranial docking approaches can be used for selective cranial or suprapubic TAR. Indications for performing a RoboTAR are medium- or large incisional hernias that require component separation to close the defect without tension and with greater mesh overlap than what is possible with retro rectus repair. It is not always clear whether component separation is needed or not. It is advisable to perform meticulous preoperative planning for all medium or large sized ventral hernias and to use CT prediction models such as the Carbonell’s equation to predict the possible need for additional TAR [35].

Apart from the complications described above, care should be taken not to damage the neurovascular bundles causing chronic pain and/or bulging or herniation of the lateral abdominal wall. This can be prevented by understanding the anatomy and use careful dissection laterally and cranially, where the neurovascular bundles enter the retromuscular space much more medially.

#### Access to the Intraabdominal Cavity and Trocar Placement

Pneumoperitoneum to 12 mm Hg is established, three trocars are placed lateral to the rectus sheath (Figure 2A) and the robot is docked. The entire abdominal wall is lysed from adhesions before starting the dissection.

**FIGURE 6:**
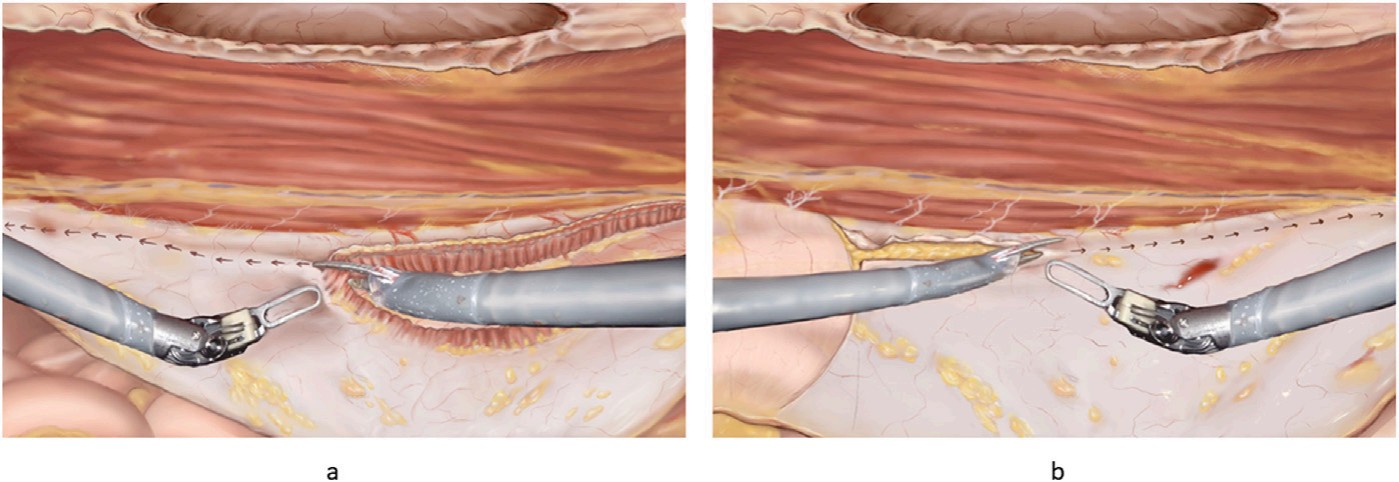
**(A)** Principles of “top down” TAR. The robotic scissors can be seen with one side lifting the fibres of the transversus abdominis. The arrows indicate the direction of incision for a full TAR. **(B)** Principles of “bottom-up” TAR. Note the fulcrum abdominalis, which is defined as the intersection between the lateral border of the rectus muscle and the arcuate line in the left part of the figure. The incision is started at the fulcrum abdominalis and continues cranially.

1. Contralateral retro rectus dissection: Incision of the medial border of the posterior sheath/fascial edges of the defect and dissection of the contralateral retro rectus is performed as described in the sections above.
2. Entering the TAR plane: This can be done either as a “top-down TAR” also called “Novitsky-way” [33] or as a “bottom-up” TAR [36].
  a. The top-down technique (Figure 6A) is performed by starting cranially at the lateral edge of the posterior sheath and incising the posterior lamella of the internal oblique 0.5–1 cm medial to the neurovascular bundles and the lateral border of the rectus sheath. The transverse abdominis (TA) muscle is then divided by using one blade of the scissors as a hook to lift the muscle off the fascia transversalis. As the TA inserts medial to the lateral border of the rectus sheath in the upper third part of the abdomen, it is easy to recognise and start the TAR here. In the cranial part of the abdomen, it is important to stay in the pre-transversalis plane right up against the muscle fibres due to the thin peritoneum. Care should be taken when dissecting the upper part of the abdominal wall and the sub-xiphoid space. In the sub-xiphoid area it is important to continue the flap dissection in the true preperitoneal plane medially and in the pre-transversalis plane laterally as described [36], making sure not to damage the neurovascular bundles and protecting the pars abdominalis of the diaphragm. It is advisable to incise the posterior sheath 6 cm below the xiphoid process in a horizontal direction entering the preperitoneal plane [30]. The entire dissection is continued downwards and laterally (see below).
  b. The bottom-up TAR technique (Figure 6B)*:* the retro rectus space is dissected completely laterally and caudally to expose the pubic bone into the space of Retzius. Care should be taken to avoid the epigastric vessels. The spermatic vessels and the vas or round ligament should be completely parietalised as described elsewhere [22]. The space of Bogros is found and the preperitoneal space is entered just below the fulcrum abdominalis: which is defined as the intersection between the lateral border of the rectus muscle and the arcuate line [24, 35]. Here the TAR plane is entered from below, and blunt dissection is continued upwards and laterally – “digging a cave” or sweeping the peritoneum and preperitoneal fat away from the aponeurotic part of the transversus abdominis before it is divided, to prevent holes in the peritoneum [20]. “Lateral to medial” dissection is another concept used to prevent holes in the posterior layer. This dissection is continued upwards until meeting the muscular part of the TA in the upper third of the abdomen, (See above).
3. Lateral extension of the TAR plane is performed by blunt dissection of the muscle fibres from the peritoneum (in the lower abdomen) and fascia transversalis (in the upper abdomen) extending the dissection laterally to the retroperitoneal fat of the flank. It is advisable to keep the transversalis fascia on the peritoneum and stay in the pre-transversalis plane in the upper part of the abdomen to avoid rents and tears in the posterior layer as the peritoneum here is thin.
4. Re-docking: When the TAR is finished on one side, three additional trocars are placed laterally to the cut edge through the dissected TA muscle, and the robot is docked from the other side (Figure 2E). Steps 2 and 3 are then performed on the other side.
5. Closure of the posterior layer: The pneumoperitoneum should be lowered to 8 mmHg to facilitate closure of the posterior sheaths using a barbed, slowly absorbable suture in a running fashion, excluding the viscera from the mesh. Tears or rents in the peritoneum are repaired with Vicryl 3-0.
6. Closing the anterior sheath: The entire linea alba and the hernia defect are closed using a continuous, running barbed suture, 1 or 0 absorbable or non-absorbable suture, as described above, taking bites into the hernia sac to reduce the dead space.
7. Mesh placement: the entire retro rectus and preperitoneal (pre-transversalis) space is measured and a fitted medium-weight macroporous permanent synthetic mesh is inserted. The large piece of mesh is folded side-to-side, keeping it together with a suture at only one side while inserting the mesh. Once inside the abdominal cavity the suture is cut and the mesh unfolds [20]. Mesh fixation is optional but can be done with fibrin glue as described above.
8. Pneumoperitoneum Release: The pneumoperitoneum is gradually released, ensuring that the mesh remains flat and properly extended without folds.

## Discussion

This article has described four of the most frequently used robotic-assisted ventral hernia repair methods in a detailed step-by-step fashion.

There are several limitations to this paper. Surgical procedures vary widely due to the large heterogeneity of the patients and hernia defect features. Furthermore, there may be several different ways of carrying out the different techniques with several personal preferences. This paper aims to describe and collect the most common tips, tricks, and technical details but is not exhaustive. The techniques can vary greatly with regards to trocar placement and dissection technique, and there are many opinions on the choice of mesh and suture products and whether to use mesh fixation but the evidence for the different choices is sparse.

Robotic ventral hernia repair has gained increasing popularity in recent years and adoption rates are rising rapidly in many high-income countries. This paper has described the four most used robotic ventral hernia procedures in a comprehensive detailed, step-by-step fashion with illustrations. However, the procedures contiously develop and improves.
