# Corrigendum: Robotic Surgical Procedures for Ventral Hernia Repair

**DOI:** 10.3389/jaws.2026.16216

**Published:** 2026-01-29

**Authors:** M. W. Christoffersen, K. Andresen, Helene Perregaard, N. A. Henriksen

**Affiliations:** 1 Department of Surgery, Zealand University Hospital Køge, Køge, Denmark; 2 Department of Surgery, Copenhagen University Hospital - Herlev and Gentofte, Herlev, Denmark; 3 Department of Surgery, Center for Perioperative Optimisation, Copenhagen University Hospital - Herlev and Gentofte, Herlev, Denmark; 4 Department of Surgery, Nordsjællands Hospital University of Copenhagen, Hillerød, Denmark

**Keywords:** robot-assisted ventral hernia repair, ventral hernia, TARM, E-TEP, RoboTAR

Masurkar AA. Laparoscopic Trans-Abdominal Retromuscular (TARM) Repair for Ventral Hernia: A Novel, Low-Cost Technique for Sublay and Posterior Component Separation. World J Surg. 2020; 44:1081–1085 was not cited in the article. The citation has now been inserted as reference 28 in the section TransAbdominal RetroMuscular Repair (TARM), paragraph 1 and should read: “Robotic-assisted placement of a mesh in the retromuscular space has different names, one of which is “Transabdominal Retromuscular Umbilical Prosthetic” (TARUP) hernia repair [26], however, the term transabdominal retromuscular repair (TARM) may be more accurate [28, 29]. The TARM repair uses the retromuscular plane laterally and the preperitoneal plane in the midline. This means that the technique involves a “cross-over” where the two retro rectus spaces are combined with the mid-preperitoneal plane posterior to the linea alba, to form one large, connected space. This approach is primarily used for midline ventral hernias, but also for more laterally placed defects, i.e., previous stoma-site hernias. Indications for this procedure include medium to large -sized (<3 cm) primary ventral hernias [23], all incisional hernias, hernias with concurrent large diastasis, multiple defects or Swiss cheese, or as a “bail out” of a TAPP repair if the peritoneum proves to be too thin or disrupted.”

The authors apologize for this error and state that this does not change the scientific conclusions of the article in any way. The original article has been updated.

